# Arthritis Among Veterans — United States, 2011–2013

**Published:** 2014-11-07

**Authors:** Louise B. Murphy, Charles G. Helmick, Kelli D. Allen, Kristina A. Theis, Nancy A. Baker, Glen R. Murray, Jin Qin, Jennifer M. Hootman, Teresa J. Brady, Kamil E. Barbour

**Affiliations:** 1 Division of Population Health, National Center for Chronic Disease Prevention and Health Promotion, CDC; 2 Health Services Research and Development Service, U.S. Department of Veterans Affairs Medical Center, Durham, North Carolina, and Thurston Arthritis Research Center, University of North Carolina at Chapel Hill; 3 Geographic Information Systems Laboratory, University of West Georgia

Arthritis is among the most common chronic conditions among veterans and is more prevalent among veterans than nonveterans (1,2). Contemporary population-based estimates of arthritis prevalence among veterans are needed because previous population-based studies predate the Persian Gulf War (1), were small (2), or studied men only (2) despite the fact that women comprise an increasing proportion of military personnel and typically have a higher prevalence of arthritis than men (1,3). To address this knowledge gap, CDC analyzed combined 2011, 2012, and 2013 Behavioral Risk Factor Surveillance System (BRFSS) data among all adults aged ≥18 years, by veteran status, to estimate the total and sex-specific prevalence of doctor-diagnosed arthritis overall and by sociodemographic categories, and the state-specific prevalence (overall and sex-specific) of doctor-diagnosed arthritis. This report summarizes the results of these analyses, which found that one in four veterans reported that they had arthritis (25.6%) and that prevalence was higher among veterans than nonveterans across most sociodemographic categories, including sex (prevalence among male and female veterans was 25.0% and 31.3%, respectively). State-specific, age-standardized arthritis prevalence among veterans ranged from 18.8% in Hawaii to 32.7% in West Virginia. Veterans comprise a large and important target group for reducing the growing burden of arthritis. Those interested in veterans’ health can help to improve the quality of life of veterans by ensuring that they have access to affordable, evidence-based, physical activity and self-management education classes that reduce the adverse effects of arthritis (e.g., pain and depression) and its common comorbidities (e.g., heart disease and diabetes).

BRFSS is an annual, cross-sectional, random-digit–dialed telephone (landline and cell phone) survey of the 50 U.S. states, territories, and the District of Columbia (DC). BRFSS is designed to collect data that are representative of the noninstitutionalized adult civilian population in each state. All analyses used combined 2011, 2012, and 2013 BRFSS data. Median state-specific BRFSS response rates, based on American Association for Public Opinion Research definition no. 4, were 49.7% in 2011, 45.2% in 2012, and 45.9% in 2013.* BRFSS respondents were defined as having arthritis if they responded “yes” to the question, “Have you ever been told by a doctor or other health professional that you have some form of arthritis, rheumatoid arthritis, gout, lupus, or fibromyalgia?” Veterans were defined as those who responded “yes” to the question, “Have you ever served on active duty in the United States Armed Forces, either in the regular military or in a National Guard or military Reserve unit? Active duty does not include training for the Reserves or National Guard, but does include activation, for example, for the Persian Gulf War.”

CDC estimated annualized crude and age-specific prevalence of doctor-diagnosed arthritis stratified by veteran status and sex, age-standardized overall and sex-specific prevalence by veteran status across categories of race/ethnicity, highest educational attainment, employment status, income, and body mass index (under/normal weight, overweight, and obese), age-standardized prevalence overall and by sex among veterans for the 50 states, DC, Guam, and Puerto Rico. Data were analyzed using software that accounted for the complex sampling design, including application of sampling weights so that estimates were representative of the noninstitutionalized adult civilian population in each state. Variance was estimated with 95% confidence intervals (CIs) that accounted for the clustered design using the Taylor series linearization method. The 2000 U.S. Projected Population, in three age groups (18–44, 45–64, and ≥65 years) was used for age-standardization.†

Veterans had a higher overall prevalence of reported arthritis than nonveterans, 25.6% (CI = 25.2%–26.1%) versus 23.6% (CI = 23.4%–23.7%). For both men and women, arthritis prevalence was higher among veterans than nonveterans (Table 1). Among male veterans (compared with male nonveterans) arthritis prevalence was higher for all age groups, and age-standardized arthritis prevalence was ≥5 percentage points higher across most of the sociodemographic categories examined (race/ethnicity, education, income, employment status, and body mass index) (Table 1). Among female veterans (compared with female nonveterans) arthritis prevalence was higher for young (18–44 years) and middle aged (44–64 years) women; age-standardized arthritis prevalence was ≥5 percentage points higher across most of the sociodemographic categories examined (Table 1). Of the estimated 9.0 million veterans with arthritis, 8.3 million were men and 670,000 were women.

Among the 50 states and DC, the median state-specific arthritis prevalence among veterans was 25.4% (range = 19.7% in DC to 32.7% in West Virginia) (Table 2, Figure). Among male veterans, the median state-specific prevalence was 24.7% (range = 18.4% in Hawaii to 32.7% in West Virginia); among women the median was 30.3% (range = 22.4% in Hawaii to 42.7% in Oregon) (Table 2). In each state, veterans comprised a substantial proportion of all persons with arthritis (median = 15.9%; range = 12.6% in Illinois and New Jersey to 22.2% in Alaska) (Table 2).

## Discussion

Veterans reported arthritis frequently and more often than nonveterans among both men and women and across all sociodemographic groups. Although a high level of physical fitness and good health are required for entry into military service, traumatic and overuse injuries are common during active duty (4). A recent study found that the incidence of osteoarthritis (a condition that represents the largest portion of arthritis cases and for which musculoskeletal injuries are a potent risk factor) was higher among an active duty sample than osteoarthritis incidence reported in civilian populations (5).

One of the few previous population-based studies of arthritis prevalence among veterans was a small study based on 2010 BRFSS data from men in five states (Indiana, Mississippi, South Carolina, West Virginia, and Wisconsin) (2). In that study, 44.8% (unadjusted) had arthritis, whereas in the current study, arthritis prevalence in these same five states was lower, ranging from 32.7% in West Virginia to 22.0% in Wisconsin. Two changes in the BRFSS methodology since 2011 might account for this difference. First, cell phone users are now sampled. Inclusion of cell phones captures younger adults who might be missed with previous landline-only data collection; the latter is more likely to capture age groups (middle aged and older adults) with a higher prevalence of arthritis. Second, sampling weights, which are applied to make estimates representative of each states’ population, are now calculated using iterative proportional fitting (raking) methods, whereas before 2011, sampling weights were derived using post-stratification procedures.§

Arthritis prevalence was consistently higher among female veterans than their male counterparts. A previously reported estimate among women using U.S. Department of Veterans Affairs (VA) health system services indicated that three in four (77.6% in 2008) had arthritis (6). Although this estimate is considerably higher than the estimate for women overall in the current study (31.3%), VA health system consumers represent a subset of veterans who are more likely to have military service–associated disability (7). In the current study, arthritis prevalence among women veterans who reported being unable to work (67.9%) was almost as high as that in the previous study. This subgroup might be most similar to VA system users.

Although the prevalence of arthritis was higher among women, the relative differences in prevalence between veterans and nonveterans was higher for men than women. Patterns across age were also noteworthy. Arthritis was not only highly prevalent among middle aged (45–64 years) veterans (40.3% among women and 36.0% among men) but also among younger veterans (prevalences of 17.3% and 11.6% among women and men aged 18–44 years, respectively) indicating that arthritis and its effects need to be addressed among male and female veterans of all ages. Reducing the impact of arthritis among younger adults might help to stem its debilitating effects in later life.

The findings in this report are subject to at least five limitations. First, arthritis was based on self-report. Although recall bias is possible, a validation study among health plan enrollees found that this definition had a positive predictive value of 74.9% among persons aged 45–64 years and a 91.0% positive predictive value among persons ages ≥65 years (8) and is acceptable for public health surveillance of arthritis. Second, there was insufficient sample size to estimate state-specific arthritis prevalence across the same sociodemographic categories as for the overall estimates (Table 1). Nevertheless, BRFSS collection of veteran status in 2011, 2012, and 2013 allowed analysis of arthritis prevalence across finer sociodemographic categories than previously possible, which was especially important in calculating sex-specific estimates. Third, similar to civilian jobs, there is considerable heterogeneity in military occupations, ranging from sedentary office jobs to physically demanding roles, including combat. BRFSS did not collect information about duration of active duty and work-related risk factors for arthritis during service (e.g., trauma/injury versus physical work demand), and therefore arthritis prevalence across these groups cannot be determined. Fourth, data are cross-sectional and not longitudinal, and therefore, attributing onset of arthritis to veteran status is not appropriate; furthermore, arthritis among veterans might be unrelated to service and attributable instead to risk factors for arthritis (e.g., obesity for osteoarthritis or smoking for rheumatoid arthritis). Finally, results might be subject to selection bias because the median BRFSS response rates were <50% in all three survey years. Nevertheless, the population-based estimates for veterans overall and across sociodemographic categories in this study demonstrate that arthritis among veterans is an important public health concern.

What is already known on this topic?

Arthritis is a common chronic condition among veterans, and at least two population-based studies have reported a higher prevalence of arthritis among veterans compared with nonveterans. These arthritis prevalence studies of veterans were conducted before the Persian Gulf War, were small, or examined men only.

What is added by this report?

To assess the prevalence of doctor-diagnosed arthritis among male and female veterans, CDC analyzed Behavioral Risk Factor Surveillance System survey data from 2011, 2012, and 2013. The analysis found that 25.6% of veterans reported having arthritis (25.0% among men and 31.3% among women) and that prevalence was higher among veterans than nonveterans across most sociodemographic categories. State-specific, age-standardized arthritis prevalence among veterans ranged from 18.8% in Hawaii to 32.7% in West Virginia.

What are the implications for public health practice?

The high prevalence of arthritis, combined with the large number of persons affected, indicate that strategies are needed to reduce the adverse effects of arthritis. Interventions to improve the quality of life of persons with arthritis include providing access to affordable physical activity and self-management education classes.

The contemporary, state-specific arthritis prevalence estimates provided in this report indicate that veterans with arthritis represented a sizeable portion (with a median of approximately one in six) of adults with arthritis in each state. Because most veterans use health systems other than the VA system (9), strategies for managing arthritis that are accessible to all veterans are essential. Fortunately, multiple self-management strategies have been proven to decrease the adverse effects of arthritis and improve the quality of life of persons with arthritis. These include courses that teach persons with arthritis how to achieve recommended levels of physical activity (e.g., Walk with Ease and EnhanceFitness)¶ and those that teach skills for better managing arthritis and other chronic conditions, including diabetes, heart disease, and chronic lung diseases (e.g., self-management education classes such as the Chronic Disease Self-Management Program).** Although these courses are increasingly available in communities across the United States, even greater availability is needed to ensure they are readily available for the large and growing number of adults with arthritis, including veterans (10). General community offerings of these programs might not appeal to some veterans or accommodate their specific needs or preferences. The high prevalence of arthritis among veterans, coupled with the large absolute number of veterans affected, suggests that dedicated veterans’ service organizations in the community and other settings are well-positioned to offer these evidence-based programs to the veteran population. Additionally, health care professionals can have a meaningful impact on improving veterans’ quality of life and function by recommending these programs to their patients with arthritis.

## Figures and Tables

**FIGURE:**
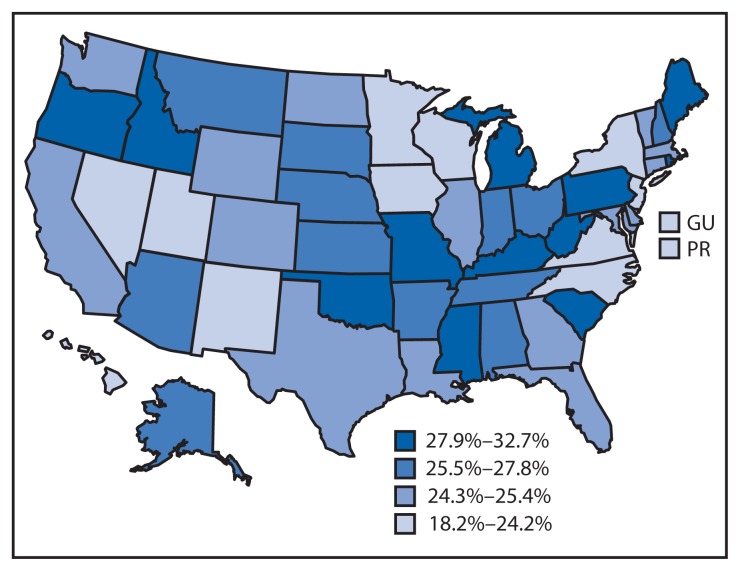
State-specific, age-standardized estimated prevalence of arthritis among veterans — United States, 2011, 2012, and 2013 Behavioral Risk Factor Surveillance System surveys **Abbreviations:** GU = Guam; PR = Puerto Rico.

**TABLE 1:** Crude, age-specific, and age-standardized* estimated prevalence of arthritis among veterans and nonveterans, by sex and selected sociodemographic characteristics — United States, 2011, 2012, and 2013 Behavioral Risk Factor Surveillance System surveys

	Sex-specific																	
		
	Men (n = 586,401)						Women (n = 875,889)						Overall (N = 1,464,060)					
			
	Nonveterans (n = 417,572)			Veterans (n = 168,829)			Nonveterans (n = 860,024)			Veterans (n = 15,865)			Nonveterans (n = 1,277,596)			Veterans (n = 111,934)		
						
Characteristic	No.†	%†	95% CI†	No.†	%†	95% CI†	No.†	%†	95% CI†	No.†	%†	95% CI†	No.†	%†	95% CI†	No.†	%†	95% CI†
**Overall**																		
Crude	98,604	17.6	(17.4 – 17.8)	66,723	35.0	(34.6 – 35.4)	324,533	28.9	(28.7 – 29.1)	6,037	31.3	(29.9 – 32.7)	423,137	24.0	(23.8 – 24.1)	72,760	34.7	(34.3 – 35.1)
Age-standardized	98,103	19.5	(19.3 – 19.7)	66,385	25.0	(24.5 – 25.4)	321,422	26.1	(26.0 – 26.3)	5,963	31.3	(29.9 – 32.7)	419,525	23.6	(23.4 – 23.7)	72,348	25.6	(25.2 – 26.1)
**Age group (yrs)**																		
18–44	12,309	6.9	(6.7–7.2)	2,473	11.6	(10.9–12.4)	24,859	9.8	(9.6–10.0)	813	17.3	(15.3–19.5)	37,168	8.4	(8.3–8.6)	3,286	12.6	(11.9–13.3)
45–64	52,662	27.4	(27.0–27.8)	19,514	36.0	(35.3–36.8)	126,332	36.8	(36.5–37.2)	2,942	40.3	(38.1–42.4)	178,994	32.7	(32.5–33.0)	22,456	36.4	(35.7–37.1)
≥65	33,132	44.5	(43.8–45.3)	44,398	47.1	(46.5–47.7)	170,231	58.2	(57.9–58.6)	2,208	58.9	(55.8–61.8)	203,363	54.6	(54.3–54.9)	46,606	47.4	(46.8–48.0)
**Race/Ethnicity** §																		
White, non-Hispanic	78,495	21.2	(21.0–21.5)	55,836	25.1	(24.6–25.7)	258,029	27.2	(27.0–27.4)	4,549	31.8	(30.2–33.4)	336,524	24.9	(24.7–25.0)	60,385	25.7	(25.2–26.2)
Black, non-Hispanic	6,934	19.5	(18.8–20.3)	4,031	25.1	(23.6–26.6)	30,127	28.1	(27.6–28.6)	738	27.7	(24.0–31.7)	37,061	24.9	(24.5–25.3)	4,769	25.8	(24.4–27.3)
Hispanic	5,536	14.3	(13.6–15.0)	2,057	21.9	(20.3–23.6)	17,350	22.7	(22.1–23.2)	245	28.8	(23.6–34.7)	22,886	18.9	(18.5–19.3)	2,302	22.7	(21.1–24.4)
Other, non-Hispanic	6,002	16.2	(15.2–17.2)	3,602	28.4	(26.4–30.4)	14,791	23.0	(22.1–23.9)	414	33.5	(28.1–39.3)	20,793	20.2	(19.6–20.9)	4,016	29.1	(27.2–31.1)
**Highest educational attainment** §																		
Less than high school	13,840	22.9	(22.3–23.6)	4,806	31.7	(28.5–35.0)	39,011	31.2	(30.7–31.8)	¶	¶	¶	52,851	27.4	(27.0–27.9)	4,941	32.9	(29.4–36.6)
High school or equivalent	31,252	20.7	(20.4–21.1)	21,041	25.0	(24.2–25.9)	110,453	27.8	(27.4–28.1)	1,163	30.1	(27.2–33.1)	141,705	25.0	(24.8–25.2)	22,204	25.3	(24.5–26.1)
Technical degree/Some college	22,770	20.4	(20.0–20.9)	19,939	26.1	(25.3–26.8)	92,571	26.7	(26.4–27.0)	2,386	33.2	(31.0–35.5)	115,341	24.5	(24.3–24.7)	22,325	26.9	(26.2–27.7)
College degree or higher	30,421	15.0	(14.7–15.3)	20,775	21.5	(20.7–22.3)	81,415	20.9	(20.7–21.2)	2,339	28.5	(26.7–30.3)	111,836	18.4	(18.3–18.6)	23,114	22.4	(21.7–23.2)
**Employment status** §																		
Working	44,285	15.7	(15.4–16.0)	16,092	20.5	(19.9–21.0)	89,980	21.3	(21.1–21.6)	1,986	24.8	(22.7–27.0)	134,265	18.7	(18.5–18.9)	18,078	20.9	(20.3–21.4)
Not working	6,261	19.3	(18.2–20.4)	2,209	27.3	(25.1–29.6)	14,569	27.7	(27.0–28.5)	326	35.6	(29.7–41.9)	20,830	24.2	(23.6–24.8)	2,535	28.2	(26.2–30.3)
Homemaker/student	791	18.6	(15.7–21.8)	291	22.5	(18.6–26.9)	33,544	22.9	(22.4–23.3)	447	30.2	(26.6–33.9)	34,335	22.2	(21.8–22.6)	738	25.8	(23.2–28.6)
Retired	31,111	33.4	(28.4–38.8)	41,535	37.3	(32.5–42.3)	136,637	33.5	(29.9–37.3)	¶	¶	¶	167,748	34.3	(31.0–37.8)	43,801	38.8	(34.3–43.5)
Unable to work	15,746	44.3	(42.9–45.8)	6,341	54.1	(50.5–57.8)	48,246	58.3	(57.2–59.4)	982	67.9	(60.6–74.5)	63,992	52.9	(52.0–53.7)	7,323	56.5	(53.2–59.8)
**Annual household income** §																		
<$15,000	13,544	25.1	(24.4–25.8)	5,274	32.7	(30.4–35.1)	53,074	34.4	(33.9–35.0)	740	42.7	(37.9–47.6)	66,618	31.0	(30.5–31.4)	6,014	33.9	(31.8–36.0)
$15,000 to <$25,000	16,443	22.5	(21.9–23.1)	11,629	30.5	(29.1–32.0)	65,049	30.0	(29.6–30.5)	1,071	35.9	(32.0–40.1)	81,492	27.1	(26.8–27.4)	12,700	31.1	(29.8–32.5)
$25,000 to <$50,000	22,202	19.5	(19.0–19.9)	19,869	25.6	(24.7–26.5)	73,142	26.5	(26.1–26.8)	1,572	31.0	(28.6–33.6)	95,344	23.7	(23.4–24.0)	21,441	26.1	(25.2–26.9)
≥$50,000	36,178	17.1	(16.8–17.4)	22,271	22.3	(21.6–22.9)	74,785	21.9	(21.6–22.2)	1,874	28.0	(25.8–30.4)	110,963	19.8	(19.6–20.0)	24,145	22.9	(22.3–23.6)
**Body mass index** §																		
Underweight/Normal weight (<25)	19,994	15.5	(15.1–15.8)	14,741	19.9	(19.1–20.7)	97,371	20.5	(20.3–20.7)	1,792	25.1	(23.0–27.3)	117,365	19.0	(18.8–19.2)	16,533	20.8	(20.1–21.6)
Overweight (25 to <30)	39,025	18.0	(17.7–18.3)	28,729	23.0	(22.3–23.6)	95,942	25.6	(25.3–25.9)	1,863	31.6	(29.2–34.2)	134,967	22.0	(21.8–22.2)	30,592	23.6	(23.0–24.3)
Obese (≥30)	38,114	26.0	(25.6–26.4)	22,537	32.4	(31.4–33.4)	109,627	35.5	(35.2–35.9)	2,039	39.9	(36.9–43.0)	147,741	31.5	(31.3–31.8)	24,576	33.0	(32.0–34.0)

**Abbreviation:** CI = confidence interval.

* Age-standardized to 2000 U.S. projected population (age groups 18–44, 45–64, and ≥65 years); includes only those for whom age was reported.

† Number of respondents (unweighted) who reported having arthritis.

§ Weighted to noninstitutionalized U.S. civilian population using sampling weights provided in Behavioral Risk Factor Surveillance System survey data.

¶ Estimates not presented if number of respondents was <50 or relative standard error was ≥30 because estimate might be unreliable.

**TABLE 2:** State-specific, age-standardized* estimated prevalence of arthritis among veterans, by sex — United States, 2011, 2012, and 2013 Behavioral Risk Factor Surveillance System surveys (N = 1,464,060)

State	Sex-specific								All veterans				Veterans with arthritis as % of all persons in state with arthritis¶

	Men				Women								
		
	No.†	No. (1,000s)§	%§	95% CI§	No.†	No. (1,000s)§	%§	95% CI§	No.†	No. (1,000s)§	%§	95% CI§	
Alabama	1,233	165	26.8	(24.4–29.2)	149	16	34.1	(28.7–39.9)	1,382	182	27.8	(25.7–30.0)	15.4
Alaska	612	24	26.6	(24.1–29.4)	65	2	26.4	(19.8–34.3)	677	26	26.6	(24.2–29.1)	22.2
Arizona	1,061	194	23.9	(21.1–27.0)	102	24	40.0	(29.7–51.2)	1,163	218	25.9	(22.9–29.2)	18.5
Arkansas	746	89	25.6	(22.5–29.0)	78	9	34.5	(26.3–43.7)	824	98	26.7	(23.8–29.8)	14.9
California	1,694	754	23.6	(21.7–25.5)	158	58	34.4	(28.9–40.4)	1,852	811	24.7	(22.9–26.6)	13.8
Colorado	1,941	141	24.7	(23.0–26.5)	176	14	31.1	(26.5–36.1)	2,117	155	25.4	(23.8–27.1)	17.7
Connecticut	905	87	24.9	(21.6–28.4)	66	5	27.6	(20.9–35.6)	971	92	25.0	(22.0–28.2)	14.1
Delaware	777	30	23.5	(20.5–26.7)	94	3	30.1	(23.4–37.7)	871	33	24.3	(21.6–27.2)	17.6
District of Columbia	420	10	19.9	(16.8–23.4)	§	§	§	§	468	10	19.7	(16.9–22.8)	10.3
Florida	3,276	639	23.8	(21.8–25.8)	313	60	34.4	(27.7–41.8)	3,589	699	25.0	(23.0–27.1)	17.5
Georgia	1,110	263	24.1	(22.0–26.3)	155	31	30.4	(25.5–35.7)	1,265	294	24.8	(22.9–26.9)	16.8
Hawaii	866	33	18.4	(16.5–20.5)	77	2	22.4	(17.6–28.2)	943	36	18.8	(17.0–20.7)	17.1
Idaho	891	50	28.9	(24.7–33.5)	76	3	30.1	(22.8–38.6)	967	53	28.7	(24.8–33.0)	18.7
Illinois	721	284	25.1	(21.4–29.3)	53	17	29.9	(22.0–39.3)	774	301	25.4	(22.0–29.1)	12.6
Indiana	1,182	171	27.3	(24.6–30.2)	90	10	31.0	(24.6–38.2)	1,272	181	27.3	(24.8–30.0)	13.3
Iowa	956	81	22.8	(20.3–25.4)	64	4	27.5	(19.4–37.4)	1,020	86	23.2	(20.8–25.9)	14.8
Kansas	2,497	80	26.2	(24.5–27.9)	223	7	33.8	(29.0–39.0)	2,720	87	26.9	(25.3–28.6)	17.2
Kentucky	1,417	134	30.2	(27.7–32.8)	133	7	29.3	(23.1–36.4)	1,550	141	30.2	(27.9–32.6)	12.9
Louisiana	1,018	117	23.4	(21.1–25.9)	88	9	31.1	(24.2–39.0)	1,106	126	24.4	(22.1–26.9)	13.7
Maine	1,678	52	28.7	(26.3–31.2)	125	3	28.1	(22.8–34.2)	1,803	55	28.5	(26.3–30.8)	17.5
Maryland	1,590	150	24.5	(22.2–27.1)	234	18	28.2	(24.2–32.6)	1,824	168	24.9	(22.8–27.1)	15.9
Massachusetts	2,159	159	23.6	(21.2–26.2)	188	12	33.1	(26.4–40.6)	2,347	171	24.9	(22.6–27.4)	13.9
Michigan	1,737	301	31.5	(28.3–34.8)	107	15	30.0	(23.5–37.5)	1,844	316	31.2	(28.3–34.2)	13.3
Minnesota	1,500	127	22.6	(20.0–25.5)	123	8	25.9	(19.5–33.5)	1,623	135	22.7	(20.2–25.4)	16.1
Mississippi	1,057	84	30.0	(26.9–33.4)	97	7	31.5	(25.2–38.5)	1,154	90	30.1	(27.2–33.1)	13.6
Missouri	1,058	190	28.4	(25.3–31.7)	86	13	33.5	(26.1–41.7)	1,144	203	28.7	(25.8–31.8)	15.3
Montana	1,585	37	26.4	(24.1–28.9)	127	3	32.0	(26.5–38.2)	1,712	40	26.9	(24.8–29.2)	19.0
Nebraska	2,946	53	25.7	(23.6–28.0)	212	4	39.5	(33.2–46.2)	3,158	57	26.8	(24.8–29.0)	17.0
Nevada	793	80	24.6	(21.2–28.2)	65	4	22.6	(17.1–29.2)	858	84	23.9	(20.9–27.1)	18.1
New Hampshire	1,077	44	28.1	(24.7–31.8)	92	3	29.2	(22.8–36.4)	1,169	48	27.8	(24.7–31.0)	17.3
New Jersey	1,524	179	21.6	(19.5–23.8)	120	10	23.8	(18.3–30.3)	1,644	190	22.0	(20.1–24.0)	12.6
New Mexico	1,225	56	23.9	(21.8–26.2)	131	5	28.1	(23.0–33.8)	1,356	61	24.2	(22.3–26.3)	16.1
New York	714	365	22.7	(20.0–25.8)	55	18	31.8	(24.4–40.1)	769	384	23.5	(20.8–26.3)	10.3
North Carolina	1,508	277	24.2	(22.3–26.2)	132	19	23.2	(18.9–28.1)	1,640	297	24.1	(22.4–25.9)	15.5
North Dakota	763	19	24.3	(21.8–27.0)	58	1	27.4	(20.6–35.4)	821	21	24.7	(22.3–27.3)	15.5
Ohio	1,566	351	26.7	(24.5–29.0)	115	20	30.9	(24.9–37.6)	1,681	372	27.2	(25.1–29.4)	14.2
Oklahoma	1,258	120	29.2	(26.6–31.9)	104	8	29.6	(24.5–35.3)	1,362	129	28.9	(26.7–31.3)	16.3
Oregon	864	120	27.6	(24.4–31.2)	93	12	42.7	(32.4–53.6)	957	133	29.1	(25.8–32.5)	16.1
Pennsylvania	2,014	384	28.4	(26.0–30.8)	159	24	35.0	(27.0–43.9)	2,173	409	29.1	(26.8–31.6)	14.1
Rhode Island	905	33	28.7	(25.3–32.5)	68	2	24.5	(18.4–31.9)	973	35	28.2	(25.0–31.6)	15.6
South Carolina	1,994	154	27.3	(25.2–29.6)	192	14	35.7	(30.5–41.2)	2,186	169	28.3	(26.3–30.3)	16.1
South Dakota	1,078	25	26.3	(22.7–30.2)	82	1	29.4	(22.8–36.9)	1,160	27	26.2	(22.9–29.7)	17.8
Tennessee	818	203	25.8	(22.2–29.7)	85	20	33.6	(24.3–44.4)	903	223	26.8	(23.4–30.4)	16.6
Texas	1,441	573	23.8	(21.7–26.0)	167	65	32.1	(25.4–39.6)	1,608	637	24.9	(22.9–27.0)	16.3
Utah	1,332	49	22.5	(20.5–24.5)	86	3	32.3	(25.4–40.0)	1,418	53	23.3	(21.4–25.3)	13.5
Vermont	891	19	24.4	(21.6–27.3)	61	1	32.8	(24.1–42.9)	952	20	25.4	(22.8–28.3)	14.8
Virginia	1,043	243	22.6	(20.7–24.6)	151	32	26.9	(22.9–31.3)	1,194	275	23.0	(21.2–24.8)	17.3
Washington	2,109	207	23.8	(22.0–25.6)	257	22	29.9	(25.4–34.8)	2,366	229	24.4	(22.8–26.1)	17.6
West Virginia	916	73	32.7	(29.8–35.8)	65	4	34.7	(27.6–42.6)	981	76	32.7	(30.0–35.6)	14.5
Wisconsin	742	154	22.0	(19.1–25.1)	55	10	28.5	(20.5–38.1)	797	164	22.4	(19.8–25.3)	14.8
Wyoming	1,054	18	24.7	(22.0–27.5)	85	1	28.1	(20.4–37.3)	1,139	20	25.0	(22.4–27.8)	18.3
*Median*			*24.7*				*30.3*				*25.4*		*15.9*
Guam	131		18.6	(15.3–22.3)	**		**	**	145		18.2	(15.2–21.6)	16.3
Puerto Rico	330		20.9	(18.0–24.1)	**		**	**	368		22.6	(19.1–26.5)	5.9

* Age-standardized to 2000 U.S. projected population (age groups 18–44, 45–64, and ≥65 years); includes only those for whom age was reported.

† Number of respondents (unweighted) who reported having arthritis.

§ Weighted to noninstitutionalized U.S. civilian population using sampling weights provided in Behavioral Risk Factor Surveillance System survey data.

¶ Number of veterans with arthritis/total number of adults in state with arthritis.

** Estimates not presented if number of respondents was <50 or relative standard error was ≥30 because estimate might be unreliable.

## Footnotes

* Additional information available at http://www.cdc.gov/brfss/annual_data/annual_data.htm.

† Additional information available at http://www.cdc.gov/nchs/data/statnt/statnt20.pdf.

§ Post-stratified weights are calculated by aligning each individual characteristic (e.g., sex and age) of the sample with the target population; iterative proportional fitting (raked weights) are calculated by iteratively aligning each specific combination of characteristics (e.g., women aged 18–25 years). Additional information available at http://www.cdc.gov/brfss/annual_data/2013/pdf/weighting_data.pdf.

¶ Additional information available at http://www.cdc.gov/arthritis/interventions/physical_activity.htm.

** Additional information available at http://www.cdc.gov/arthritis/interventions/self_manage.htm.
